# *Morinda officinalis* oligosaccharides mitigate chronic mild stress-induced inflammation and depression-like behaviour by deactivating the MyD88/PI3K pathway *via* E2F2

**DOI:** 10.3389/fphar.2022.855964

**Published:** 2022-08-16

**Authors:** Zhen-Hua Zhu, Xu-Yuan Yin, Tu-Sun Xu, Wei-Wei Tao, Guang-Da Yao, Pei-Jie Wang, Qi Qi, Qiu-Fang Jia, Jing Wang, Yue Zhu, Li Hui

**Affiliations:** ^1^ Research Center of Biological Psychiatry, Suzhou Guangji Hospital, Medical College of Soochow University, Suzhou, China; ^2^ Jiangsu Collaborative Innovation Center of Chinese Medicinal Resources Industrialization, National and Local Collaborative Engineering Center of Chinese Medicinal Resources Industrialization and Formulae Innovative Medicine, Nanjing University of Chinese Medicine, Nanjing, China; ^3^ Ningxia Medical University, Ningxia Key Laboratory of Cerebrocranial Disease, Incubation Base of National Key Laboratory, Nanjing, China

**Keywords:** depression, inflammation, MyD88/PI3K, E2F2, morinda officinalis oligosaccharides

## Abstract

*Morinda officinalis* oligosaccharides (MOs) are natural herbal extracts that have been shown to exert antidepressant effects. However, the mechanism of this effect remains unclear. Here, we explored the mechanism by which MOs improved experimental depression. Using a chronic mild stress (CMS) murine model, we examined whether MOs could protect against depressive-like behaviour. Lipopolysaccharide (LPS)- and ATP-treated BV2 cells were used to examine the potential mechanism by which MOs mediate the inflammatory response. We found that MOs prevented the CMS-induced reduction in the sucrose preference ratio in the sucrose preference test (SPT) and shortened the immobility durations in both the tail suspension test (TST) and forced swim test (FST). We also noticed that MOs suppressed inflammatory effects by deactivating the MyD88/PI3K pathway *via* E2F2 in CMS mice or LPS- and ATP-stimulated BV2 cells. Furthermore, overexpression of E2F2 blunted the beneficial effects of MOs *in vitro*. Collectively, these data showed that MOs exerted antidepressant effects in CMS mice by targeting E2F2-mediated MyD88/PI3K signalling pathway.

## 1 Introduction

Major depressive disorder (MDD), which is also known as depression, is a mental disease characterized by a wide range of symptoms, including depressed mood, cognition dysfunction, and abnormalities in appetite and sleep (Fava and Kendler, 2000). MDD is a seriously disabling public health problem with a very high prevalence. Approximately 350 million people suffer from depression, leading to great societal and economic burdens worldwide (Athira et al., 2020). At present, mainstream antidepressants to treat depression act through monoaminergic mechanisms (Belujon and Grace, 2017). Unfortunately, 2/3 of patients do not achieve remission after one course of treatment, and 1/3 fail to remit after four treatments (Rush et al., 2006). Therefore, it is urgent to develop new effective drugs for the treatment of depression.

Inflammation is the result of immune system activation, which often manifests as a localized reaction resulting from irritation, injury, or infections (Beurel et al., 2020). The brain possesses specialized immune cells called microglia that are activated by various stimuli. Cytokines participate in the induction and effector phases of inflammatory responses and are predominantly produced by immune cells, including microglia (Beurel et al., 2020). Previous evidence has shown that inflammation plays a vital role in depression. Patients with depression show an increased inflammatory response (Howren et al., 2009; Ye et al., 2018). Higher levels of inflammation increase the risk of the development of depression (Nowak et al., 2019). Furthermore, antidepressant therapies have been reported to inhibit inflammatory effects (Kalkman and Feuerbach, 2016).

The PI3K/AKT pathway belongs to the family of serine/threonine protein kinases and is implicated in the regulation of several downstream target proteins, including NF-κB, through the phosphorylation of IκBα and p65 (Ju et al., 2020). This pathway is involved in inflammation and depression. Abnormalities in this pathway have been found in inflammation-mediated depression induced by lipopolysaccharide (LPS) (Guo et al., 2019; Sun et al., 2021). Vilazodone, a novel antidepressant, improves the depressed mood of MDD patients, which is associated with reduced NF-κB activity (Eyre et al., 2017). Increased phosphorylation levels of PI3K, AKT, and p65 induced by notoginsenoside R1 exert significant antidepressant efficacy in CUMS rats by alleviating the inflammatory response in the hippocampus (Zhan et al., 2022). MyD88 is an essential component of TLR signalling that binds to PI3K and influences the PI3K/AKT/NF-κB pathway (Gelman et al., 2006; Laird et al., 2009; Wang S. et al., 2018). The interactions between MyD88 and PI3K have been observed by using an immunoprecipitation assay (Laird et al., 2009). Gelman et al. found that the MyD88 death domain is required for NF-κB activation, and MyD88 mutations abolish the association of MyD88 and PI3K, as well as the phosphorylation of AKT, in T cells (Gelman et al., 2006). Another study reported that E2F2 controls the PI3K/AKT/NF-κB axis by binding to MyD88 (Wang S. et al., 2018).

*Morinda officinalis* oligosaccharides (MOs) are bioactive compounds extracted from the roots of this plant. MOs have been approved by the China Food and Drug Administration (CFDA) for use as a prescribed traditional herbal medicine to treat depression (Li et al., 2021). Significant attenuation of behavioural deficits was found in rodents exposed to chronic unpredictable stress after treatment with MOs (Xu et al., 2017). However, the mechanism underlying MO-related antidepressant effects has not been fully elucidated. Recently, MOs have been shown to suppress hippocampal inflammation in poststroke rats (Li et al., 2021). Therefore, in the current study, we investigated whether MOs mitigated depression by targeting hippocampal inflammation by using a chronic mild stress (CMS) mouse model of depression and a lipopolysaccharide (LPS)- and adenosine triphosphate (ATP)-induced cellular model of inflammation.

## 2 Materials and methods

### 2.1 Animals and treatment

Male C57BL/6 mice (10–12 weeks of age) were used in this study. The mice were housed under a constant temperature of 23 ± 1°C with a 12-h light and dark cycle and free access to food and water. All animal experiments were approved by the Institutional Review Board at the Affiliated Guangji Hospital of Soochow University.

The mice were randomly assigned to five groups (12 per group): 1) control group; 2) CMS + saline group (model); 3) CMS + 25 mg/kg MO group (MO-L); 4) CMS + 50 mg/kg MO group (MO-H); and 5) CMS +20 mg/kg fluoxetine group (Flu). Mice in the CMS groups were housed individually and exposed to two random stressors per day for 7 weeks. The stressors included food deprivation (24 h), water deprivation (24 h), cage tilting (24 h), damp bedding (24 h), inversion of the day/night light cycle, restraint in a tube (2 h), and tail clipping (1 min). MOs (Tongrentang) and fluoxetine (Sigma–Aldrich) were administered (i.g.,) once daily for three consecutive weeks starting in week 5.

### 2.2 Cell culture and treatment

Murine BV2 microglial cells were obtained from the Cell Bank of the Type Culture Collection of the Chinese Academy of Sciences. The cells were maintained in Dulbecco’s modified Eagle’s medium (DMEM) containing 10% foetal bovine serum (FBS) and 1% penicillin–streptomycin solution in a humidified 5% CO_2_ atmosphere at 37°C. BV2 cells were pretreated with MOs (2.5, 5, 10 mg/ml) for 24 h and then treated with LPS (10 μg/ml) and ATP (5 mM) for another 12 h (Supplementary Figure S1).

For the E2F2 overexpression experiment, BV2 cells were transfected with vectors overexpressing E2F2 (Supplementary Figure S2) or empty vectors using Lipofectamine 2000 (Thermo Fisher Scientific) according to the manufacturer’s protocol.

### 2.3 Luciferase reporter assay

The wild-type (WT) or mutant (MUT) MyD88 promoter was cloned into a pGL3-basic vector (Promega). Then, HEK239T cells were cotransfected with either pcDNA3.1 or pcDNA3.1-E2F2 using Lipofectamine 2000 according to the manufacturer’s instructions. Luciferase activity was determined using a Dual Luciferase Assay (Promega).

### 2.4 Sucrose preference test

The SPT was performed to assess anhedonia in mice according to a published procedure (Shen et al., 2020). Briefly, all mice were exposed to two bottles of 1% sucrose for 48 h, followed by 24 h of water deprivation. On the testing day, the animals were provided a pre-weighed bottle of 1% sucrose and a pre-weighed bottle of drinking water for 2 h (Shen et al., 2020). The positions of the two bottles were switched at the midway point of the test to avoid side preferences. At the end of the test, the bottles were weighed, and sucrose preference was calculated as the percentage of sucrose solution intake divided by the total fluid intake for each mouse.

### 2.5 Tail suspension test

The TST was performed according as previously described (Meng et al., 2020). Each mouse was suspended by the tail from a vertical bar for 6 min, and the total immobility time of the final 4 min of the 6-min testing period was analysed by ANY-MAZE software. The animals were judged to be immobile when they hung passively and ceased moving their limbs and body.

### 2.6 Forced swim test

The FST was conducted as previously described (Zhu et al., 2020). Each mouse was individually placed in a plastic cylinder (diameter: 30 cm; height: 40 cm) containing 25 cm of water at 24 ± 1°C and allowed to swim for 6 min. The immobility time was recorded during the final 4 min of the 6-min test. Mice were judged to be immobile when they made only minimal movements to keep their head above water.

### 2.7 Open field test

The OFT was performed as reported previously (Sevastre-Berghian et al., 2020). The mice were placed in the centre of the open field arena and allowed to freely explore the area for 5 min. Their movements were recorded, and the total distance travelled and time spent in the central area were analysed by ANY-MAZE software.

### 2.8 Quantitative real-time PCR (qRT–PCR)

Total RNA was extracted from hippocampi or cells using TRIzol reagent (Invitrogen) according to the manufacturer’s protocols. Quantitative RNA analysis was performed using Nanodrop spectrophotometry. After reverse transcription of total RNA into cDNA, SYBR Green qPCR Master Mix was used for real-time PCR detection according to the manufacturer’s instructions. The expression of target genes was normalized to GAPDH mRNA levels. The primers used in this study are listed below.

**Table:** 

Primers	Forward	Reverse
E2F2	TCGCAGAGACCATAGAGCCT	GGATTGGGGACAGGAACTGG
MyD88	AGGCATCACCACCCTTGAT	ATTAGCTCGCTGGCAATGGA
TNF-α	AGGCACTCCCCCAAAAGATG	CCACTTGGTGGTTTGTGAGTG
IL-1α	CAACGTCAAGCAACGGGAAG	CAAACTTCTGCCTGACGAGC
IL-1β	GAAATGCCACCTTTTGACAGTGA	GTCCTCATCCTGGAAGGTCC
GAPDH	GGGTCCCAGCTTAGGTTCATC	TACGGCCAAATCCGTTCACA

### 2.9 ELISA

The levels of TNF-α (ab208348, Abcam), IL-1α (SEKM-0001, Solarbio) and IL-1β (ab197742, Abcam) in serum and cells were measured by ELISA using commercially available kits according to the manufacturer’s protocol.

### 2.10 Immunofluorescence

Immunofluorescence staining was performed as previously reported (Lyu et al., 2018). Sections or cells were fixed in 4% paraformaldehyde for 20 min, permeabilized with 0.5% Triton X-100 for 20 min, and blocked with 3% BSA for 1 h. Then, the samples were incubated with primary antibodies against Iba1 (ab178847, Abcam, 1:100), E2F2 (AF4100, Affinity, 1:100) and p-NF-κB p65 (AF 2006, Affinity, 1:200) overnight at 4°C, followed by incubation with goat anti-rabbit IgG Alexa Fluor® 488 (ab150077, Abcam, 1:200) for 1 h at room temperature. Cell nuclei were labelled with 4′-6-diamidino-2-phenylindole (DAPI), and images were obtained with a confocal microscope (Olympus Fluoview TM1000).

### 2.11 Western blotting

Western blotting was performed as previously described (Ning et al., 2014). Total protein was extracted from hippocampi or cells using RIPA lysis buffer and quantified by a bicinchoninic acid protein assay kit. Equal amounts of protein were then subjected to SDS-PAGE and transferred onto PVDF membranes. After being blocked with 5% nonfat milk for 1 h, the blots were probed with primary antibodies against E2F2 (AF4100, Affinity, 1:1,000), MyD88 (DF6162, Affinity, 1:1,000), p-PI3K (ab182651, 1:1,000, Abcam), PI3K (ab191606, Abcam, 1:1,000), p-AKT (ab38449, Abcam, 1:1,000), AKT (ab8805, Abcam, 1:500), p-NF-κB p65 (AF 2006, Affinity, 1:1,000), NF-κB p65 (AF5006, Affinity, 1:1,000) and GAPDH (ab22555, Abcam, 1:3,000) overnight at 4°C. The corresponding secondary antibodies (ab6728, Abcam, 1:4,000) were added and incubated for 1.5 h at room temperature. Immunoreactive bands were visualized by an enhanced chemiluminescence reagent and quantified by NIH ImageJ software.

### 2.12 MTT assay

Cell viability was determined by the MTT assay (Cui et al., 2020). Cells were plated into 96-well plates and treated with different concentrations of MOs for 24 h. Subsequently, 20 μL of MTT (5 mg/ml) was added to the wells. After incubation for 4 h at 37°C, the formazan was dissolved in dimethyl sulfoxide (DMSO). Optical density (OD) values were measured at a wavelength of 570 nm by a microplate reader.

### 2.13 Statistical analysis

The data are presented as the means ± SD. The results were analysed using GraphPad Prism 6.0 software. Significant differences were determined by Student’s t test or analysis of variance (ANOVA). Differences of *p* < 0.05 were considered statistically significant.

## 3 Results

### 3.1 Activation of MyD88/PI3K signaling in chronic mild stress-exposed mice

**FIGURE 1:**
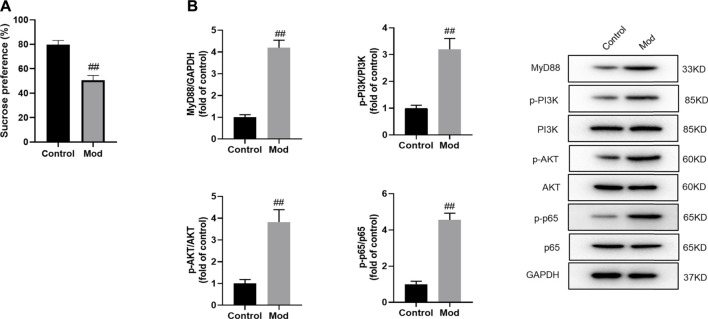
Protein expression of MyD88, p-PI3K, p-AKT and p-NF-κB p65 in the hippocampus of CMS-exposed mice. **(A)** Mice were subjected to the CMS protocol for 4 weeks, then sucrose preference test was performed. **(B)** Densitometric analysis and representative western blots of MyD88, p-PI3K, p-AKT and p-NF-κB p65 expressed in the hippocampus. Data are expressed as means ± SD (*n* = 3). #*p* < 0.05; ##*p* < 0.01.

First, we examined the changes of the MyD88/PI3K pathway in the hippocampus of CMS-exposed mice. The sucrose pereference ratio was significantly decreased in stressed mice over the control group, suggestive of successful CMS modeling (Figure 1A). It is noteworthy that CMS mice exhibited higher MyD88 expression and phosphorylation levels of PI3K, AKT and NF-κB p65 than nonstressed controls (*p* < 0.01), which suggested the activation of MyD88/PI3K signalling in depression (Figure 1B).

### 3.2 *Morinda officinalis* oligosaccharides alleviate depressive behaviour and inflammation by suppressing the E2F2-mediated MyD88/PI3K pathway in chronic mild stress-exposed mice

**FIGURE 2:**
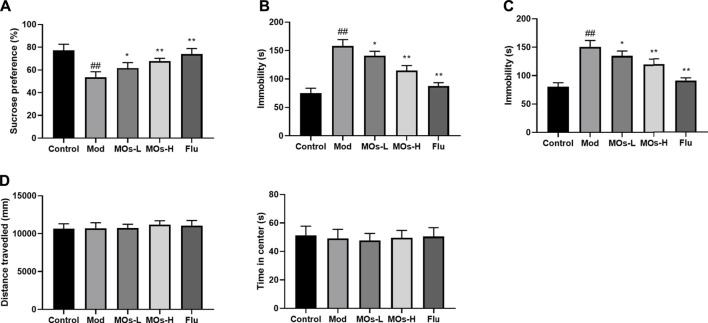
MOs block CMS-induced depressive-like behavior. **(A)** Mean sucrose preference (%) in the sucrose preference test. **(B)** The duration of immobility during the tail suspension test. **(C)** The duration of immobility during the forced swim test. **(D)** Total distance travelled and time spent in the center zone in the open field test. Data are expressed as means ± SD (*n* = 10). ##*p* < 0.01 vs. control; *p < 0.05; **p < 0.01 vs. Mod; ns, not significant.

To investigate whether MOs alter depressive behaviour in CMS-exposed mice, we performed the SPT, TST and FST (Figures 2A–C). The mice that underwent the CMS challenge exhibited decreases in sucrose preference in the SPT and increases in immobility durations in the TST and FST (*p* < 0.01), and these effects were attenuated by MOs or Flu administration (*p* < 0.05, *p* < 0.01), indicating the antidepressant efficacy of MOs and Flu in CMS-induced depression. However, no differences were detected in the OFT test (*p* > 0.05, Figure 2D).

**FIGURE 3:**
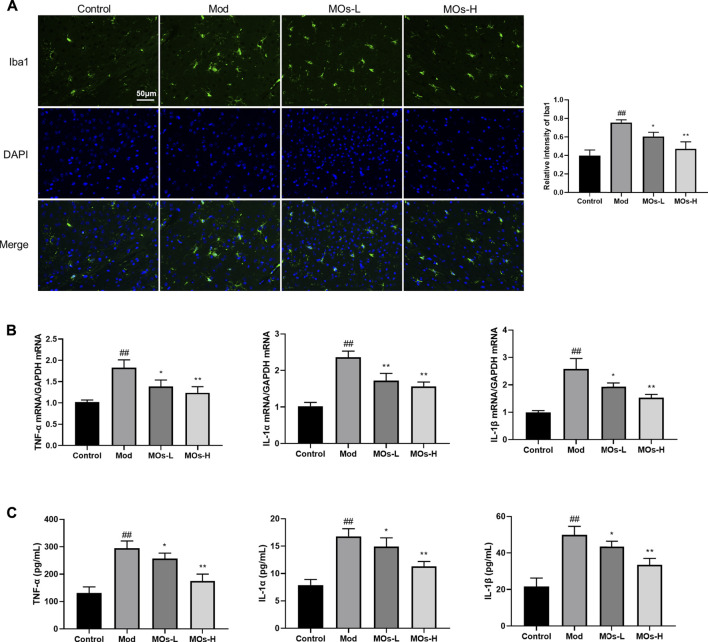
MOs protect from CMS-induced inflammation. **(A)** Representative hippocampus immunofluorescent staining for Iba1 (green) and nuclei (blue), and quantification of relative intensity of Iba1 (*n* = 3). **(B)** Measurement of TNF-α, IL-1α and IL-1β mRNA levels in the hippocampus (*n* = 3). **(C)** TNF-α, IL-1α and IL-1β concentrations in the serum (*n* = 6). Data are expressed as means ± SD. ##*p* < 0.01 vs. control; *p < 0.05; **p < 0.01 vs. Mod.

**FIGURE 4:**
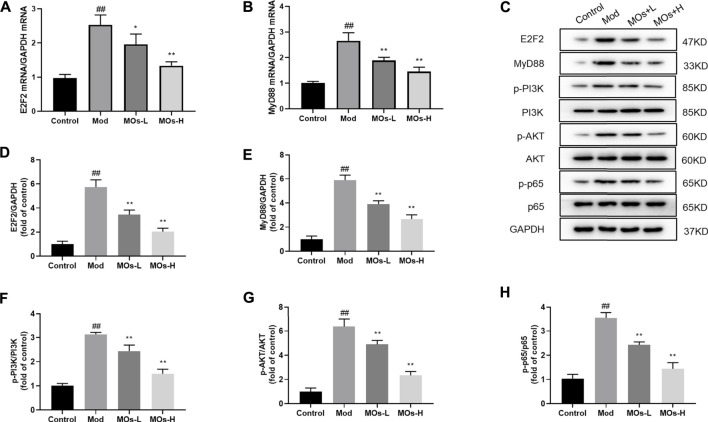
Effects of MOs on E2F2-regulated MyD88/PI3K pathway in the hippocampus. **(A–B)** qRT-PCR analysis of gene expression of E2F2 and MyD88 in hippocampus tissues. **(C–H)** Immunoblot analysis of E2F2, MyD88, p-PI3K, p-AKT and p-NF-κB p65 in hippocampus tissues. Data are expressed as means ± SD (*n* = 3). ##*p* < 0.01 vs. control; *p < 0.05; **p < 0.01 vs. Mod.

Next, we evaluated inflammation in the mice. Since abnormal activation of microglia, the immunologic guardian cells of the brain, is a major contributor to depression-related inflammation, we measured Iba1 (a key marker of microglia) fluorescence intensity to assess microglial activation (Brites and Fernandes, 2015; Wang Y.-L. et al., 2018). The stressed mice exhibited significant increases in Iba1 immunoreactivity (Control: 0.401 ± 0.0594, Mod: 0.754 ± 0.0293, MOs-L: 0.604 ± 0.0451, MOs-H: 0.471 ± 0.0751), concomitantly with enhanced levels of proinflammatory cytokines, including TNF-α (PCR: Control: 1.02 ± 0.053, Mod: 1.83 ± 0.186, MOs-L: 1.39 ± 0.154, MOs-H: 1.24 ± 0.144; ELISA: Control: 132 ± 22.0 pg/ml, Mod: 294 ± 26.8 pg/ml, MOs-L: 256 ± 20.4 pg/ml, MOs-H: 175 ± 25.2 pg/ml), IL-1α (PCR: Control: 1.02 ± 0.103, Mod: 2.36 ± 0.168, MOs-L: 1.72 ± 0.199, MOs-H: 1.57 ± 0.117; ELISA: Control: 7.90 ± 1.00 pg/ml, Mod: 16.8 ± 1.38 pg/ml, MOs-L: 14.9 ± 1.58 pg/ml, MOs-H: 11.3 ± 0.880 pg/ml) and IL-1β (PCR: Control: 0.994 ± 0.0635, Mod: 2.58 ± 0.384, MOs-L: 1.93 ± 0.139, MOs-H: 1.53 ± 0.118; ELISA: Control: 21.7 ± 4.64 pg/ml, Mod: 49.9 ± 4.69 pg/ml, MOs-L: 43.5 ± 2.95 pg/ml, MOs-H: 33.5 ± 3.56 pg/ml), suggesting the activation of microglia after CMS modelling (*p* < 0.01, Figures 3A–C). However, these effects were suppressed by MO treatment (*p* < 0.05, *p* < 0.01). Furthermore, PCR and western blotting revealed that the mRNA expression of E2F2 and MyD88 and the protein levels of E2F2, MyD88, p-AKT, p-NF-κB p65 and p-PI3K were upregulated by CMS exposure (*p* < 0.05) and downregulated by MO intervention (*p* < 0.05, *p* < 0.01, Figures 4A–H). Together, these findings showed that MOs mitigated CMS-induced depressive behaviour and inflammation by deactivating E2F2-controlled MyD88/PI3K signalling in mice.

### 3.3 *Morinda officinalis* oligosaccharides inhibit E2F2 binding to MyD88

**FIGURE 5:**
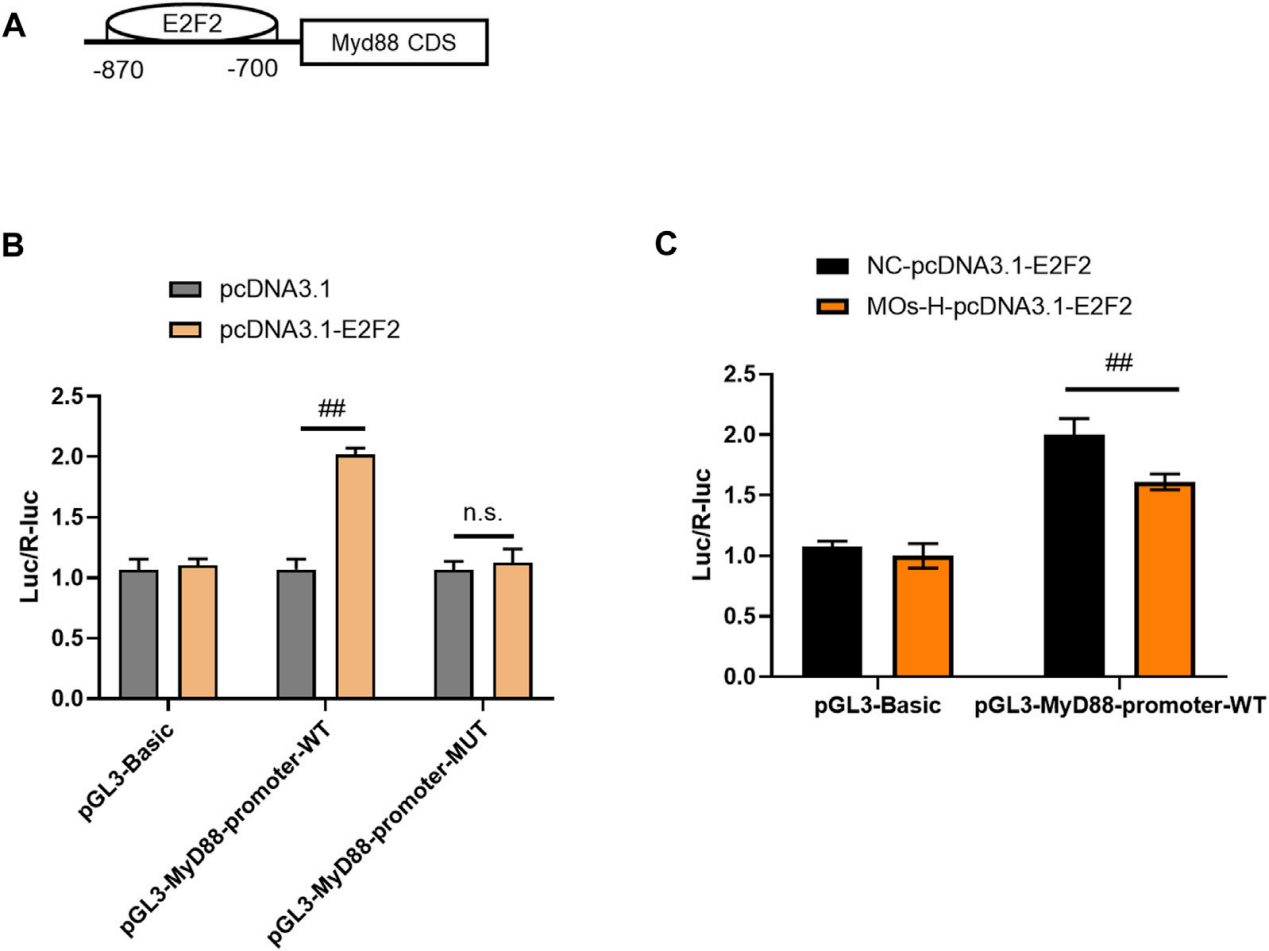
MOs inhibit E2F2 binding to MyD88 in HEK239T cells. **(A)** Schematic representation of the E2F2 binding motif in the MyD88 promoter. **(B)** Luciferase reporter gene assay of E2F2 and MyD88. **(C)** Effects of MOs (50 mg/kg) on E2F2 binding to MyD88 in the HEK239T cells. Data are expressed as means ± SD (*n* = 3). ##*p* < 0.01; n. s. not significant.

To validate the interaction between MyD88 and E2F2, luciferase reporter assays were performed in HEK293T cells (Figures 5A–C). Cotransfection of the MyD88-promoter-WT plasmid and E2F2 vector markedly increased luciferase expression compared with the effect of the MyD88-promoter-WT plasmid lacking the E2F2 vector (*p* < 0.01). However, cotransfection of the MyD88 promoter-MUT plasmid and E2F2 vector did not affect luciferase activity (*p* > 0.05). These findings showed that E2F2 could directly bind to the promoter of the MyD88 gene. We then assessed the effect of MOs on this binding process. Surprisingly, MO treatment inhibited the binding of E2F2 and the MyD88 promoter, as demonstrated by lower luciferase expression in the MyD88-promoter-WT + MO-H-E2F2 group than in the MyD88-promoter-WT + NC-E2F2 group (*p* < 0.01).

### 3.4 *Morinda officinalis* oligosaccharides protect against lipopolysaccharide- and adenosine triphosphate-induced inflammation *via* E2F2-mediated MyD88/PI3K signalling in BV2 cells

**FIGURE 6:**
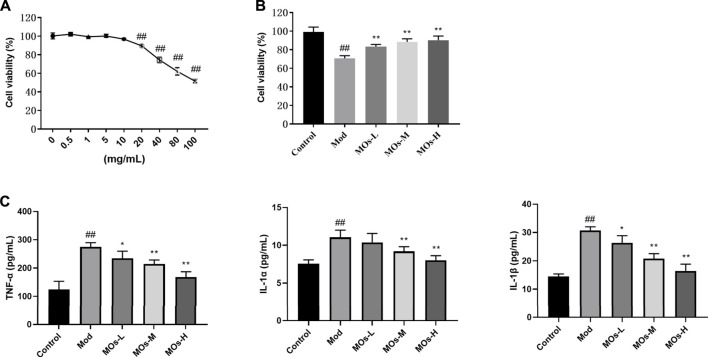
Protective effects of MOs against LPS- and ATP-induced inflammation in BV2 cells. **(A)** BV2 cells were administrated with different concentrations of MOs for 24 h, and the cell viability was detected using the MTT assay. **(B)** Cell viability assay of BV2 cells after 24 h of MOs treatment followed by LPS and ATP stimulation. **(C)** ELISA assays to determine the production of TNF-α, IL-1α and IL-1β in BV2 cells. Data are expressed as means ± SD (*n* = 6). ##*p* < 0.01 vs. control; *p < 0.05; **p < 0.01 vs. Mod.

**FIGURE 7:**
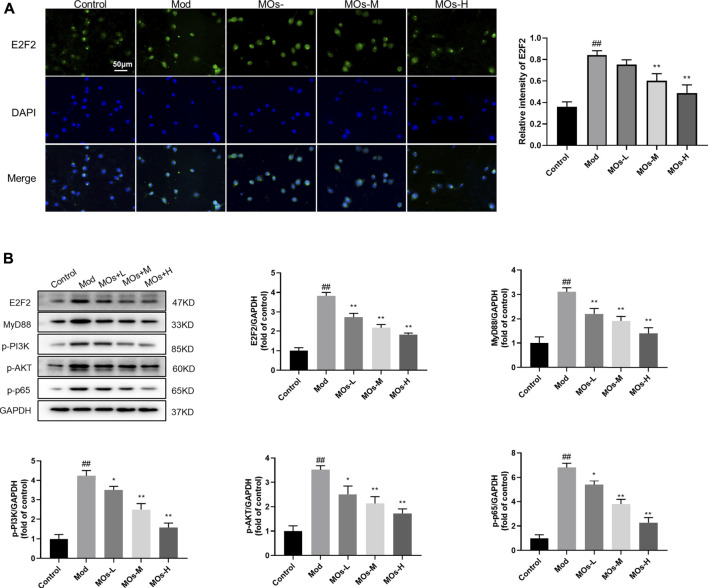
Effects of MOs on E2F2-controlled MyD88/PI3K pathway in LPS- and ATP-stimulated BV2 cells. **(A)** Representative hippocampus immunofluorescent staining for E2F2 (green) and nuclei (blue), and quantification of relative intensity of E2F2. **(B)** Protein expression levels of E2F2, MyD88, p-PI3K, p-AKT and p-NF-κB p65 in BV2 cells. Data are expressed as means ± SD (*n* = 3). ##*p* < 0.01 vs. control; *p < 0.05; **p < 0.01 vs. Mod.

We also examined the protective effect of MOs in LPS- and ATP-induced cellular models of inflammation. Given that the maximum concentration of MOs that exerted no cytotoxicity in BV2 cells was 10 mg/ml, this dose was selected as the high dose for use in subsequent experiments (Figure 6A). As expected, MOs prevented LPS- and ATP-induced reductions in cell viability and the promotion of TNF-α, IL-1α and IL-1β levels (*p* < 0.05, *p* < 0.01, Figures 6B,C). Subsequently, we examined the effect of MOs on the E2F2-mediated MyD88/PI3K signalling pathway (Figures 7A,B). The results showed that MOs decreased E2F2 immunoreactivity and the protein expression of E2F2, MyD88, p-PI3K, p-AKT and p-NF-κB p65 in LPS- and ATP-exposed BV2 cells (*p* < 0.05, *p* < 0.01). Thus, MOs exerted anti-inflammatory effects *via* the E2F2-regulated MyD88/PI3K pathway *in vitro*.

**FIGURE 8:**
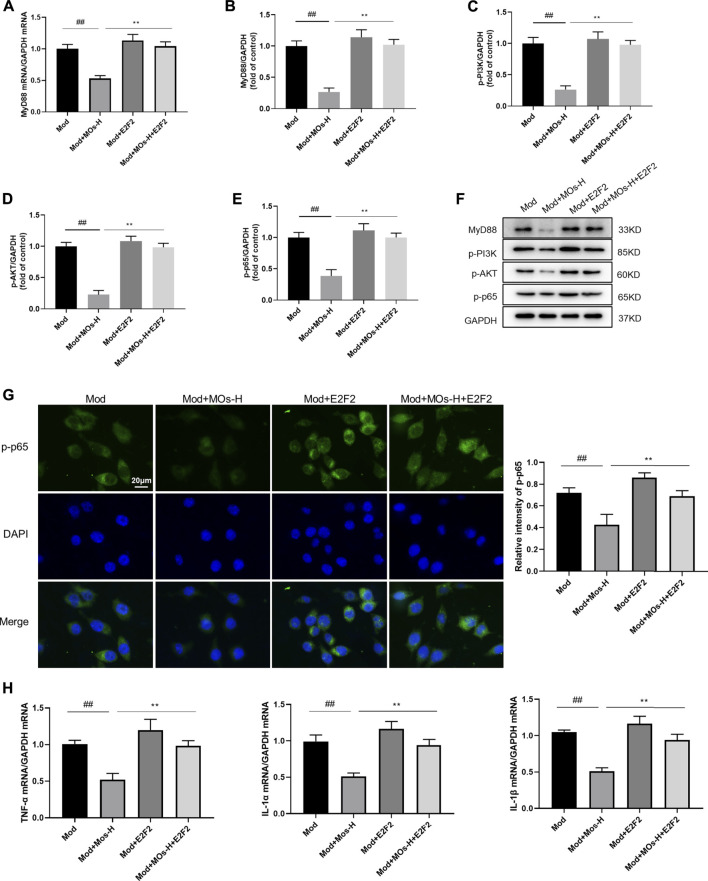
Effects of MOs in LPS- and ATP-stimulated BV2 cells were reversed by overexpression of E2F2. **(A)** qRT-PCR analysis of MyD88 mRNA expression in BV2 cells. **(B–F)** Immunoblot analysis of MyD88, p-PI3K, p-AKT and p-NF-κB p65 in BV2 cells. **(G)** Representative hippocampus immunofluorescent staining for p-NF-κB p65 (green) and nuclei (blue), and quantification of relative expression of p-NF-κB p65. **(H)** TNF-α, IL-1α and IL-1β mRNA levels in BV2 cells. Data are expressed as means ± SD (*n* = 3). ##*p* < 0.01; **p < 0.01.

To further elucidate the role of E2F2 in the anti-inflammatory effect of MOs, BV2 cells were transfected with E2F2 overexpression vectors (Figures 8A–H). We found that E2F2 overexpression reversed MO-induced decreases in MyD88 mRNA expression; MyD88, p-PI3K, p-AKT and p-NF-κB p65 protein expression; and p-NF-κB p65 immunoreactivity (*p* < 0.01), resulting in increased mRNA levels of proinflammatory factors (TNF-α, IL-1α and IL-1β, *p* < 0.01). These findings suggested that E2F2 inhibition was required for MO-induced anti-inflammatory effects *in vitro*.

## 4 Discussion

In the current study, we investigated the inflammatory mechanism related to the antidepressant activity of MOs. We found that MOs ameliorated the depressive-like symptoms in mice underwent CMS protocol. MOs also inhibited CMS- or LPS + ATP-induced high levels of inflammation by targeting the MyD88/PI3K signaling pathway *via* E2F2. Additionally, overexpression of E2F2 reversed MO-produced anti-inflammatory effect *in vitro*.

Depression is a common psychiatric disorder associated with marked suffering. Since synthetic antidepressants have obvious disadvantages, such as limited efficacy, side effects and high prices, the antidepressant properties of natural medicines are attracting increasing attention (Liu et al., 2015). Feng et al. reported that Bupleuri Radix attenuated depression-like behaviour in rats by regulating metabolic profiles and the gut microbiota (Feng et al., 2020). A double-blind, randomized clinical trial revealed that crocin extracted from saffron (*Crocus sativus* L.) mitigated depressive symptoms in patients with breast cancer during chemotherapy (Salek et al., 2021). In the FST, the antidepressant efficacy of silexan, an essential oil from the flowering tops of *Lavandula angustifolia*, was comparable to the tricyclic antidepressant imipramine after 9 days of treatment (Friedland et al., 2021). In the current study, we investigated the antidepressant activity of the herbal medicine MOs by using a chronic mild stress (CMS) mouse model. CMS is one of the most widely used rodent models of depression. The primary variable measured in CMS is sucrose preference; stressed mice show reductions in sucrose consumption, which is interpreted as anhedonia, a core symptom of depression (Ramaker and Dulawa, 2017). Here, we found that MOs inhibited the reduction in sucrose consumption in the sucrose preference test and increased immobility durations in the tail suspension and forced swim tests, indicating the antidepressant activities of MOs in depressive rodents and further supporting the potential use of natural medicines in treating depression. It is noteworthy that although CMS rodents have been reported to have abnormalities such as decreased duration in the central zone in the OFT test, we did not observe any changes in the OFT like some other studies, which might be associated with differences in CMS protocols (Zhou et al., 2019; Shan et al., 2020; Xia et al., 2020).

Inflammation is an essential immune response that enables survival during infection or injury and maintains tissue homeostasis under a variety of noxious conditions (Medzhitov, 2010). Studies have indicated that inflammation regulates a wide variety of diseases, including depression. Compared with healthy controls, patients with major depression have exhibit increased TNF-α and IL-1β levels both in the cerebrospinal fluid and in the peripheral blood circulation (Raison et al., 2006). The administration of interferon (IFN)-α (a potent inducer of proinflammatory cytokines) to treat cancer results in the development of depressive symptoms in a high percentage of patients (Capuron et al., 2002). In addition, antidepressants, such as selective serotonin reuptake inhibitors (SSRIs), exert negative immunoregulatory effects, suppressing the release of proinflammatory cytokines, such as IFN-γ, TNF-α, IL-1β and IL-6, and stimulating the generation of anti-inflammatory cytokines, such as IL-10 (Xia et al., 1996; Maes et al., 1999). In this study, we found that the CMS protocol increased Iba1 expression as well as TNF-α, IL-1α, and IL-1β levels in the hippocampus or serum, while MOs successfully inhibited this effect in CMS-exposed mice, leading to the attenuation of depression-like symptoms. These findings confirmed the involvement of inflammation in the aetiology of depression and MO-induced antidepressant efficacy.

The MyD88/PI3K pathway is an important pathway for regulating inflammation and depression. MyD88 is an adapter protein that mediates signal transduction for most TLRs. Previous evidence suggests that MyD88 can bind to the lipid kinase enzyme PI3K, which phosphorylates downstream target AKT and enhances inflammatory response *via* the NF-κB signal (Laird et al., 2009; Shorning et al., 2020). Patients with major depression have higher levels of IL-6 than healthy controls (Wang et al., 2019). Aerobic exercise inhibits MyD88/NF-κB signalling and hippocampal inflammation, which contributes to improvements in desperate behaviour and hippocampal function (Qu et al., 2020). LPS enhances the levels of IL-1β, IL-6, and TNF-α in the hippocampus and evokes depressive-like behaviours in mice, and these effects are alleviated by baicalin through the PI3K/AKT pathway (Guo et al., 2019). Astrocyte-specific reductions in Men1 levels enhance NF-κB activation and IL-1β production, leading to the development of depression in mice, and these effects can be rescued by an NF-κB inhibitor (Leng et al., 2018). In this work, upregulated MyD88 mRNA expression and enhanced phosphorylation of PI3K, AKT and NF-κB p65 were observed following CMS modelling. However, MO intervention greatly normalized the mRNA expression and protein levels of the MyD88/PI3K axis, reflecting the importance of this signalling pathway in MO-induced anti-inflammatory effects on depression. Furthermore, E2F2 overexpression blocked the MO-induced anti-inflammatory effect on LPS- and ATP-induced BV2 cells, confirming the necessary role of E2F2 in MO-mediated antidepressant activities. Previously, MOs have been demonstrated to generate antidepressant activities in rodents by suppressing hippocampal inflammation through the microglial NLRP3 inflammasome (Li et al., 2021). Here, we observed that hippocampal MyD88/PI3K signalling was also an important pathway by which MOs attenuated hippocampal inflammation and ultimately induced antidepressant effects. In addition, the PI3K signalling was activated by E2F2 binding to the MyD88 promoter in the present study, which was consistent with the results observed by Wang et al. in rheumatoid arthritis (Wang S. et al., 2018).

There are certain limitations to the study. First, we only assessed the antidepressant effect of MOs in the CMS model, and other models, such as the LPS-induced depression model, should be used in future studies to fully clarify the antidepressant efficacy of MOs. Second, although the *in vitro* results indicated the important role of E2F2 in MO-induced antidepressant effects, animal studies with E2F2-overexpression vectors might be needed to elucidate the role of E2F2 in MO-mediated antidepressant effects. Third, conventional antidepressants have a major disadvantage in their several-week-long lag period of therapeutic efficacy, and whether MOs can exert a rapid onset antidepressant effect is worth further exploration.

In summary, our data demonstrated that MOs alleviate experimental depression and inflammation *via* the E2F2-mediated MyD88/PI3K signalling pathway. This work provides a promising molecular agent for the treatment of depression.

## Data Availability

The raw data supporting the conclusions of this article will be made available by the authors, without undue reservation.
