# Supplementary material for: Morinda officinalis oligosaccharides mitigate chronic mild stress-induced inflammation and depression-like behaviour by deactivating the MyD88/PI3K pathway via E2F2
Source: Front Pharmacol. 2022 Aug 16;13:855964. doi: 10.3389/fphar.2022.855964 (PMC9426723; doi:10.3389/fphar.2022.855964)
Supplement: Supplementary file 1 [file DataSheet2.pdf]

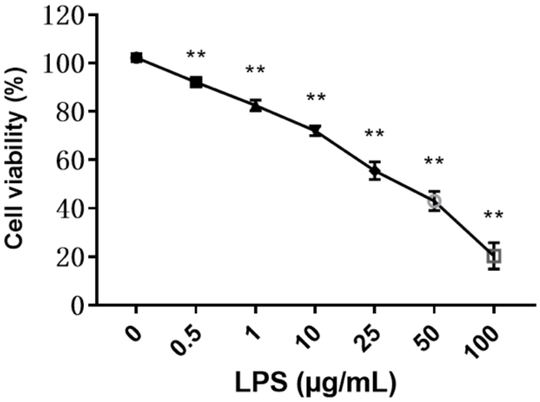

Fig. S1. Cell viability in LPS- and ATP-treated BV2 cells.
10μg/mL LPS combined with 5 mM ATP treatment reduced the cell viability to around 70%, thus this concentration of LPS was used in further experiments. Data are expressed as means ± SD (n = 6). **P < 0.01 vs control group.

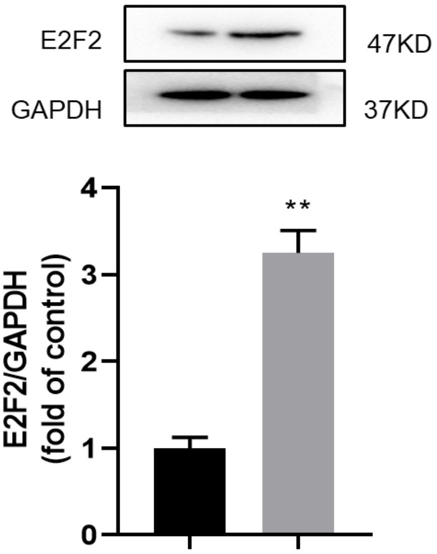

Fig. S2. Transfection efficiency of vectors overexpressing E2F2.
Protein levels determined by the western blot analysis. Data are expressed as means ± SD (n = 3). **P < 0.01.
