# Supplementary material for: Morinda officinalis oligosaccharides mitigate chronic mild stress-induced inflammation and depression-like behaviour by deactivating the MyD88/PI3K pathway via E2F2
Source: Front Pharmacol. 2022 Aug 16;13:855964. doi: 10.3389/fphar.2022.855964 (PMC9426723; doi:10.3389/fphar.2022.855964)
Supplement: Supplementary file 2 [file DataSheet1.PDF]

Fig. 1

MyD88/GAPDH

Control	Mod
0.8961	4.0039
1.1265	4.0056
0.9773	4.5911

p-PI3K/PI3K

Control	Mod
1.0817	2.7366
1.045	3.4408
0.8731	3.4275

p-AKT/AKT

Control	Mod
0.7936	3.3318
1.0987	3.7021
1.1079	4.4435

p-p65/p65

Control	Mod
1.1605	4.248
1.0008	4.4394
0.8383	4.9665

Fig. 2

SPT

Control	Mod	MOs-L	MOs-H	Flu
80.6	58.8	61.4	66.4	69.1
79.3	49.8	67.1	72.4	74.3
73.6	58.7	57.3	66.9	71.6
70.6	55.1	57.8	65.8	78.4
75.4	49.5	58	65.8	65.6
73.4	59.6	57.5	68.3	82.9
79.3	55.7	69.3	67.6	76
86.8	54.4	65.3	71.9	71.1
70.6	49.1	65.9	66.5	73.7
82.6	46.3	55.4	66.2	76.7

TST

Control	Mod	MOs-L	MOs-H	Flu
70.7	171.4	152.9	111.8	83.2
70.6	170.5	134.1	110.5	86
74.7	146.1	137	113.9	81.6
71.2	154.9	130.5	120	93.3
86.7	151.5	140.8	106.4	98
88.1	149	145.5	105.2	86.9
66.4	163.2	134.1	121.7	94.7
85.9	148.6	154.1	107.6	80.7
72	151.5	141.7	133.8	82.3
65.7	175.6	136.5	117.3	86.8

FST

Control	Mod	MOs-L	MOs-H	Flu
85.2	147.5	139.9	108.8	94.2
72.1	132.6	129.5	123.2	91.2
82	155.1	133.1	108.8	85.9
85	158.9	144.2	115.1	90.6
83.5	133.9	143	127.4	91.6
70.5	142.5	143.7	122.7	86.1
80.7	150.6	124.7	106.2	95.8
88.4	161.8	129.2	134.4	91.1
87.2	163.1	139.6	127.7	83.1
69.7	159.1	121.3	120.6	97.8

OFT-distance

Control	Mod	MOs-L	MOs-H	Flu
10968	10581	10012	11278	11467
10035	9862	11325	11245	10637
10060	11413	9898	10576	10609
11606	10357	10606	10149	10046
9817	10032	10465	11741	11814
10702	10029	11160	11299	9984
10124	10251	11007	11129	11989
10954	11675	10602	11962	11449
10872	11239	11067	11092	11410
11562	11746	11229	11405	11053

OFT-time

Control	Mod	MOs-L	MOs-H	Flu
56.7	53.8	45.4	54.7	57.5
51.7	47.5	40.1	40.8	53
42.9	53.6	44	46.3	55.8
44.8	44.6	42.9	52.9	41.8
44.8	59.9	45.9	58.4	55
43.8	42.6	56.4	49.1	45.3
55.9	49.7	49.9	47	46.8
55.2	42.7	47.4	43.6	48.8
57.8	41.9	51.7	50.3	42.2
59.1	55.7	52.7	51.9	58.4

Fig.3

Iba1

Control	Mod	MOs-L	MOs-H
0.4265	0.721	0.6538	0.5558
0.3332	0.7637	0.5658	0.4118
0.4436	0.7772	0.5925	0.4467

TNF-α mRNA

Control	Mod	MOs-L	MOs-H
1.07039	1.610915	1.212213	1.132716
1.01862	1.931876	1.435205	1.401946
0.96436	1.935125	1.508001	1.178364

IL-1α mRNA

Control	Mod	MOs-L	MOs-H
1.07047	2.172071	1.512444	1.431422
0.90545	2.485937	1.906503	1.645504
1.09376	2.432252	1.751682	1.620841

IL-1β mRNA

Control	Mod	MOs-L	MOs-H
0.93214	2.342372	1.771278	1.495126
1.05908	2.369398	1.966807	1.429478
0.99197	3.021248	2.03926	1.658964

serum TNF-α

Control	Mod	MOs-L	MOs-H
122.2174	265.1739	220.3478	168.6522
163.2174	259.8261	280.1304	184.6087
122.7391	295.7826	263.1304	155.4783
154.6522	324.7826	248.7391	142.6522
108.8261	316.8696	260.5652	213.913
117.5217	304.3913	266	185.1739

serum IL-1α

Control	Mod	MOs-L	MOs-H
9.766169	15.47264	15.9403	12.27612
7.554726	17.08458	16.14677	9.992537
7.121891	14.6791	15.04478	10.59453
6.99005	17.75124	16.45025	12.02985
7.925373	17.93035	13.06965	11.27861
8.014925	17.80597	12.89552	11.72139

serum IL-1β

Control	Mod	MOs-L	MOs-H
22.74219	51.51563	44.01563	32.67969
17.28906	53.28906	43.40625	40.60156
16.70313	54.22656	37.96094	33.00781
21.71094	42.61719	44.45313	30.98438
21.92188	45.55469	46.70313	32.71094
29.57813	52.21094	44.625	31.22656

Fig.4

E2F2 mRNA

Control	Mod	MOs-L	MOs-H
0.90804	2.298494	1.888143	1.226907
0.92595	2.854544	2.2914	1.458032
1.09098	2.426783	1.698488	1.305776

MyD88 mRNA

Control	Mod	MOs-L	MOs-H
1.08162	2.299523	2.009738	1.311156
0.96172	2.899836	1.892886	1.412066
0.97842	2.767053	1.761773	1.643654

E2F2/GAPDH	Control	Mod	MOs-L	MOs-H
	0.8745	5.486	3.3354	1.9561
	0.844	5.3092	3.1293	1.7798
	1.282	6.4205	3.8741	2.3506

MyD88/GAPDH	Control	Mod	MOs-L	MOs-H
	0.8926	5.7518	3.8495	2.8024
	0.8064	5.5609	3.6647	2.2814
	1.3011	6.3565	4.2111	2.9286

p-PI3K/PI3K	Control	Mod	MOs-L	MOs-H
	1.1116	3.1439	2.7086	1.7083
	0.9232	3.2058	2.3962	1.4501
	0.9652	3.0488	2.2119	1.3404

p-AKT/AKT	Control	Mod	MOs-L	MOs-H
	1.3274	7.0421	5.2754	2.3905
	0.9115	6.2429	4.7383	2.6253
	0.7616	5.8122	4.6723	1.9983

p-p65/p65	Control	Mod	MOs-L	MOs-H
	1.2298	3.741	2.4942	1.7122
	0.9852	3.6031	2.516	1.414
	0.8853	3.2919	2.2947	1.2098

Fig.5

Luc/R-luc(B)

group		pcDNA3.1			pcDNA3.1-E2F2	
pGL3-Basic	0.9	1.1	1.2	1.01	1.2	1.1
pGL3-MyD88-WT	1.1	0.91	1.2	2	1.94	2.12
pGL3-MyD88-MUT	0.94	1.18	1.08	0.92	1.16	1.3

Luc/R-luc(C)

group		NC-pcDNA3.1-E2F2			MOs-H-pcDNA3.1-E2F2	
pGL3-Basic	1.03	1.12	1.08	1	1.1	0.9
pGL3-MyD88-WT	2.12	1.86	2.03	1.62	1.54	1.67

Fig.6

Cell viablility (A)	0	0.5	1	5	10	20	40	80	100
	96.96878768	101.7006414	99.81657652	99.0841104	96.89330298	90.121862	73.2025757	65.1435815	53.6136543
	98.09142004	102.2558879	98.8367151	100.3351892	97.2354361	88.7292666	71.162551	67.1110007	50.391404
	101.5840671	101.1986177	99.83696107	98.12990208	97.99481078	91.1878011	79.1203484	59.7359877	53.6186623
	100.5694939	101.9390916	99.66606064	100.4198251	96.75208683	87.366036	76.9536638	56.4856847	51.1087432
	98.4232692	102.8388233	98.83623424	101.9905511	97.4890531	89.6576917	74.0501038	60.2001804	49.9011532
	105.8482311	102.8891254	99.45474926	100.9550603	94.72084848	90.7405122	72.262331	64.4105163	50.4974932

Cell viablility (B)	Control	Mod	MOs-L	MOs-M	MOs-H
	103.130643	74.00736698	82.25959774	88.45632217	96.56418552
	99.34953835	70.03198747	85.55943609	86.09817078	84.90334092
	91.40889419	74.39501707	82.12530142	84.48533636	86.85739962
	105.3935461	67.59509559	79.75930783	93.09592618	89.88149486
	93.95529028	69.1700258	84.89089216	91.7457015	94.317196
	100.7620881	69.19783688	85.73326379	86.63756714	89.15798716

TNF-α	Control	Mod	MOs-L	MOs-M	MOs-H
	146.8696	274.5217	218.3043	212.8261	148.6087
	86.6087	282.4348	212.5217	201.3913	195.7391
	109.087	247.1739	263.087	202.6957	146.1739
	167.9565	286.2174	207.3913	239.4783	178.7391
	122.4783	272.2174	259.3478	219.7391	172.7826
	116.4348	287.2174	248.8696	212.3913	167.6522

IL-1α	Control	Mod	MOs-L	MOs-M	MOs-H
	8.559701	10.59204	10.60945	8.179104	9.21393
	7.355721	10.04975	12.07463	9.09204	8.067164
	7.368159	10.05473	10.77612	9.972637	7.604478
	7.380597	11.57711	8.728856	9.723881	7.5
	7.664179	12.301	9.355721	9.099502	7.870647
	7.156716	11.75124	10.77114	9.069652	7.79602

IL-1β

Control	Mod	MOs-L	MOs-M	MOs-H
15.22656	31.17969	25.17969	21.83594	15.29688
15.14063	32.46094	29.67188	22.74219	14.75781
15.23438	31.625	24.61719	20.64063	14.625
13.17188	29.73438	29.5	22.05469	20.71094
14.50781	28.96094	25	18.11719	15.51563
13.57031	30.50781	24.19531	19.10938	17.86719

Fig.7

E2F2 relative intensity

Control	Mod	MOs-L	MOs-M	MOs-H
0.307518	0.886772	0.782075	0.632201	0.520731
0.3975	0.8288	0.7026	0.5274	0.4037
0.3744	0.808	0.7748	0.648	0.5427

E2F2/GAPDH

Control	Mod	MOs-L	MOs-M	MOs-H
1.1745	4.0188	2.9551	2.3139	1.9186
0.9565	3.6841	2.6683	2.2189	1.7904
0.8692	3.7817	2.5604	1.9896	1.7707

MyD88/GAPDH

Control	Mod	MOs-L	MOs-M	MOs-H
1.2821	3.2673	2.4487	2.0968	1.6678
0.8009	2.9301	2.1493	1.916	1.3193
0.9173	3.1209	2.0127	1.7285	1.2218

p-PI3K/GAPDH

Control	Mod	MOs-L	MOs-M	MOs-H
1.245	4.5491	3.6983	2.1537	1.6976
0.9465	4.1207	3.3321	2.7507	1.7225
0.8084	4.0524	3.4653	2.581	1.3252

p-AKT/GAPDH

Control	Mod	MOs-L	MOs-M	MOs-H
1.2355	3.6549	2.8909	2.4443	1.9225
0.9498	3.3328	2.3527	2.0335	1.5497
0.8149	3.5755	2.2761	1.9042	1.6905

p-p65/GAPDH

Control	Mod	MOs-L	MOs-M	MOs-H
0.8543	7.1664	5.4312	3.7971	2.216
1.3263	6.8056	5.6699	4.1867	2.7172
0.819	6.4482	5.0275	3.4213	1.8903

Fig.8

MyD88 mRNA

Mod	Mod+MOs	Mod+E2F2	Mod+MOs+E2F2
1.0054	0.582129	1.226846	1.11239
1.06822	0.512643	1.135918	0.974969
0.93209	0.495244	1.033275	1.040867

MyD88/GAPDH

Mod	Mod+MOs	Mod+E2F2	Mod+MOs+E2F2
1.0779	0.3285	1.2701	1.0975
1.0105	0.2702	1.1197	1.0368
0.9116	0.2064	1.0329	0.9337

p-PI3K/GAPDH

Mod	Mod+MOs	Mod+E2F2	Mod+MOs+E2F2
1.1095	0.2896	1.1989	1.0586
0.9232	0.1951	0.9789	0.9393
0.9673	0.3052	1.0325	0.9373

p-AKT/GAPDH

Mod	Mod+MOs	Mod+E2F2	Mod+MOs+E2F2
0.9395	0.2955	0.9967	0.9128
0.9957	0.2271	1.1322	1.0079
1.0649	0.1625	1.1257	1.0342

p-p65/GAPDH

Mod	Mod+MOs	Mod+E2F2	Mod+MOs+E2F2
1.0847	0.464	1.2266	1.0764
0.9926	0.4227	1.1038	0.9878
0.9225	0.2744	1.0096	0.9401

p-p65 relative intensity

Mod	Mod+MOs	Mod+E2F2	Mod+MOs+E2F2
0.7432	0.5136	0.8771	0.7081
0.7509	0.3248	0.81	0.7312
0.6647	0.4373	0.8935	0.6379

TNF-α mRNA

Mod	Mod+MOs	Mod+E2F2	Mod+MOs+E2F2
0.95155	0.530692	1.115749	1.055782
1.051	0.601751	1.367799	0.979153
1.02096	0.428321	1.116298	0.913719

IL-1α mRNA

Mod	Mod+MOs	Mod+E2F2	Mod+MOs+E2F2
0.9338	0.45720499	1.243396	0.854779
0.94776	0.537857546	1.199196	0.94761
1.09412	0.539784585	1.052254	1.014756

IL-1β mRNA

Mod	Mod+MOs	Mod+E2F2	Mod+MOs+E2F2
1.06399	0.457205	1.243396	0.854779
1.01539	0.537858	1.199196	0.94761
1.0661	0.539785	1.052254	1.014756

Fig. S1

LPS+ATP

0	0.5	1	10	25	50	100
100.3546353	93.82338819	82.03520245	72.5635274	59.87788388	48.5728484	18.6952966
101.4594165	90.07860951	84.0925768	69.89097224	51.00777843	41.6986714	13.5379728
102.6918003	91.77897385	81.33134513	75.58632074	58.80144549	39.1243124	29.7026545
103.9718065	93.81410185	79.48216555	71.05684582	51.68070456	46.1036923	17.7776337
104.4080991	92.66514081	85.77086908	71.94351832	55.19272864	38.6929944	20.0017702
100.9044473	90.53757516	82.40088655	71.17515486	56.99597972	44.2347106	22.4824636

Fig. S2

E2F2/GAPDH

Control	E2F2
0.882	2.9593
1.1306	3.4592
0.9872	3.3239
